# De-identification of primary care electronic medical records free-text data in Ontario, Canada

**DOI:** 10.1186/1472-6947-10-35

**Published:** 2010-06-18

**Authors:** Karen Tu, Julie Klein-Geltink, Tezeta F Mitiku, Chiriac Mihai, Joel Martin

**Affiliations:** 1 Institute for Clinical Evaluative Sciences (ICES) G106, 2075 Bayview Avenue, Toronto, Ontario, M4N 3M5, Canada; 2 Department of Family and Community Medicine-University of Toronto, 263 McCaul Street, 5th Floor Toronto, Ontario, M5T 1W7, Canada; 3 Toronto Western Hospital Family Health Team-University Health Network, 399 Bathurst Street, Toronto, Ontario, M5T 2S8, Canada; 4 Institute for Information Technology, National Research Council, 1200 Montreal Road, Ottawa, Ontario, K1A 0R6, Canada

## Abstract

**Background:**

Electronic medical records (EMRs) represent a potentially rich source of health information for research but the free-text in EMRs often contains identifying information. While de-identification tools have been developed for free-text, none have been developed or tested for the full range of primary care EMR data

**Methods:**

We used *deid *open source de-identification software and modified it for an Ontario context for use on primary care EMR data. We developed the modified program on a training set of 1000 free-text records from one group practice and then tested it on two validation sets from a random sample of 700 free-text EMR records from 17 different physicians from 7 different practices in 5 different cities and 500 free-text records from a group practice that was in a different city than the group practice that was used for the training set. We measured the sensitivity/recall, precision, specificity, accuracy and F-measure of the modified tool against manually tagged free-text records to remove patient and physician names, locations, addresses, medical record, health card and telephone numbers.

**Results:**

We found that the modified training program performed with a sensitivity of 88.3%, specificity of 91.4%, precision of 91.3%, accuracy of 89.9% and F-measure of 0.90. The validations sets had sensitivities of 86.7% and 80.2%, specificities of 91.4% and 87.7%, precisions of 91.1% and 87.4%, accuracies of 89.0% and 83.8% and F-measures of 0.89 and 0.84 for the first and second validation sets respectively.

**Conclusion:**

The *deid *program can be modified to reasonably accurately de-identify free-text primary care EMR records while preserving clinical content.

## Background

The uptake of electronic medical records (EMRs) is increasing amongst family physicians in Canada and around the world[1,2]. EMRs contain comprehensive clinical information regarding the course of care including lab results, prescriptions, patient risk factors, family history and past medical history in addition to many physical measures such as height, weight, blood pressure and detailed information on clinical encounters not presently available from other data sources. However, EMRs were not designed for research but rather to help physicians improve their clinical practice. As such, secondary use of this data is impeded by the fact that much of the rich clinical data contained in EMRs is not entered in a format that lends itself easily to analysis[3]. Specifically, the lack of methods for de-identifying the narrative free-text portions of EMR data in order to preserve privacy has presented a major challenge for researchers interested in utilizing this data.

At the Institute for Clinical Evaluative Sciences (ICES) we have developed an **E**lectronic **M**edical **R**ecord **A**dministrative data **L**inked **D**atabase (EMRALD) using data from family physician EMRs. This EMR data is linked through unique scrambled health card numbers to the multiple health related administrative databases for the province of Ontario, housed at ICES. ICES is an independent, not-for-profit health services research organization with a unique designation as a 'prescribed entity' in Section 45(1) of the *Personal Health Information Protection Act *(PHIPA), Ontario's privacy legislation[4]. This means that ICES has policies and procedures in place to protect the privacy and confidentiality of patients[5] as required by the Act (s.45(3)), which have been reviewed and approved by the Information and Privacy Commissioner of Ontario. This status allows ICES to receive and use health information without consent for the purposes of analysis and compiling statistical information about our health care system. Even though ICES does not release any individual level information, a free-text de-identification tool is needed in order to further enhance privacy measures through all steps of in-house EMR data analysis.

Although a number of software programs have been developed to address the issue of de-identification of narrative free-text for different types of medical data, [6-17] none have been customized for the full range of primary care EMR notes. These notes contain free-text from a wide variety of sources including point form progress notes, consultation letters from different practitioners in a variety of specialties, diagnostic test results, pathology reports and hospital discharge summaries. These free-text records use a wide variety of formatting and syntax, making it more complex to devise a tool.

Approaches to free-text de-identification include machine-learning based systems[11,13] or lexicon and pattern-based systems[6-8,10,15-17]. The machine-learning systems use labeled examples to automatically search for a statistical pattern of indicator features. For example, a human annotator would label U.S. zip codes or Canadian postal codes as elements to remove from EMRs. Then, features from the text such as the capitalization pattern, the appearance of digits, the term itself, the part of speech and syntactic dependencies are used to find a statistical rule that distinguishes between the postal codes and other text. Success in de-identifying medical discharge summaries has been achieved using a support vector machine (SVM) as the machine-learning algorithm[11]. In this case, the SVM attempts to find a separating hyperplane between the positive (labeled) and negative examples where the examples are described using a specified set of text-based features.

On the other hand, the lexicon and pattern approach uses a manually (instead of automatically) built collection of word lists, regular expressions, and heuristics. This second approach has the disadvantage that experts must spend time to create and organize the word lists and patterns. However, this characteristic can also be an advantage because the expert can include knowledge of the field that goes beyond the available training examples or beyond a fixed set of local features.

It is possible to adapt either type of system, but the style of adaptation differs. Adaptation of a machine-learning based system emphasizes adding additional training examples and modifying the set of text-based features. This adaptation would require expertise to label the new examples and then would require a large number of iterations to evaluate the effect of different features. Given that we are regularly adding EMR records from clinics in different geographic locations that receive information from different institutions and specialty areas, the adaptation of a lexicon and pattern system[17] emphasizing extending word lists, adding new word lists and adding and removing regular expressions appeared to be more appropriate for our needs. For the most part, new words and patterns can be added independently of each other such that the effects of a change are predictable to the expert. This type of adaptation can require more time from the expert, but again presents the possibility of quickly introducing additional domain knowledge without having to constantly retrain the system each time a new clinic is introduced.

Most of the work done previously in this area has been designed to de-identify all personal health information (PHI) as outlined by the Heath Insurance Portability and Accountability Act (HIPAA) in the United States. While PHI such as names and locations are not necessary to preserve, PHI such as age, dates of hospitalizations, procedures and visits have clinical implications which are important to preserve in EMR data in order to fully utilize the data for research and evaluation purposes.

We set out to determine if *deid*, [17] an open source software program designed and tested on hospital nursing notes, could be modified to de-identify primary care EMR records in EMRALD with high precision and while preserving clinically important content.

## Methods

### Initial name removal

The EMRALD database has been developed using data from family physicians in Ontario using Practice Solutions^® ^EMR, which is owned and operated by the Canadian Medical Association, and is the leading EMR software vendor in Ontario with approximately 50% of the market share of government funding supported EMRs[18]. All clinically relevant data fields from volunteering family physician's using Practice Solutions^® ^EMR for at least two years are extracted through an automated 'plug-in' triggered by the physician or their designate. Structured names and address fields are not extracted and the data goes through an initial name removal as part of the extraction process. This name removal is based on the patient name and family physician name captured in structured fields. The program searches for the occurrence of the name in the free-text data and replaces it with a randomly generated fake gender-specific first and last name. This preliminary de-identification does not remove all names, as names of family members, nicknames and misspelled names, or names of physicians or other healthcare providers that are working outside the clinic are not removed. Next the extracted data is encrypted and transmitted securely and electronically to ICES. Immediately upon arrival at ICES, data covenanters partition off the health card numbers to be scrambled for linkage to administrative data. Other identifying information such as date of birth, gender and postal code are stripped and kept in files separated from the main bulk of the data.

### Creating a reference standard

Free-text fields in the EMR include all fields in the cumulative patient profile (history of past health, active problems, family history and allergies), progress notes generated at each physician visit, referral letters, consultation letters and diagnostic tests. A random sample of 1000 free-text notes from all the different types of free-text fields from a group practice with over 10,000 patients was used as a training set for the modified *deid*. We pulled an additional two sets of free-text notes to serve as validation sets. One set was comprised of 700 notes from 17 physicians located in 7 different clinics distributed throughout Southern Ontario, while another had 500 notes from a group practice located in a geographic location that was different then the location of the practice used in the training set.

Free-text fields were manually 'tagged' for patient and physician names, hospitals and other healthcare facilities/clinics, street names, Ontario cities, businesses, health card numbers, postal codes, phone and medical record numbers, websites and email addresses, by one of the study staff. All of the tagged records were run through the program which generated a list of words that were removed, false positives and false negatives. The lists were reviewed in detail and any word that appeared to be incorrectly removed by the program was reviewed by using a simple word search function to identify where it appeared in the text. If a tagging error was made the tag was corrected. This process was repeated several times for each data set until we believed there were no more tagging errors. These corrected tagged records served as the reference standard for evaluating the performance of the modified program.

The original *deid *program and the modified *deid *program were run on the training records in an incremental fashion. First it was run on 500 free-text training records, tests of accuracy were performed, false positives and negatives were reviewed, further modifications were made to the program and then it was tested on the original 500 plus an additional 250 training records, a similar process was repeated and then the program was run on the full 1000 free-text training records.

Once the final modified *deid *program was optimized to achieve the best results possible on the training data, the program was run on the two validation sets to assess the validity and generalizability of the newly modified program.

### Deid Program Modifications

#### Dates

In order to preserve clinical context and to allow for linkage to the administrative databases at ICES that records dates and reasons for hospitalization and billing dates of physician clinical encounters, we disabled the date removal functionality in the *deid *software to preserve dates recorded in the EMR. Since birth dates are captured and stored elsewhere in the database, we removed date of birth from the free-text fields by performing a separate search for date of birth based on the information captured in the structured date of birth field.

In addition, in order to avoid having the program erroneously recognize months of the year as someone's name, all months that were written in text format were changed to number format (ie. June 1, 2007 was changed to 06/01/2007).

#### Assessing the original deid program

The *deid *program works by scanning the medical text line by line and parsing the text into individual words. The program identifies PHI by using lists and regular expressions. PHI that involve numeric patterns, such as street addresses or telephone numbers, are identified by regular expressions based on numeric patterns as well as appearances of context words such as "road" for street address or "pager" for pager number. In the case of non-numeric patterns, like names and locations, dictionary look-ups and context are used to locate both known and potential PHI. Next, the program performs pattern matching using regular expressions that look for patterns with various context keywords to find more named entities. Simple heuristics are used to qualify or disqualify ambiguous terms as PHI. Finally, each PHI is replaced with a tag denoting its corresponding category. After changing the date functionality of the program we ran the rest of the original *deid *program on the first 500 notes from the training set of free-text records.

#### Locations, healthcard and medical record numbers

The pre-existing *deid *software uses lists based on American context, thus these lists were replaced with Ontario lists for street names,[19] municipalities,[20] healthcare facilities[21] and businesses[22]. Healthcare facilities were separated and grouped according to type in order to replace the facility with another similar type to preserve clinical context. Pharmacies and insurance companies and business names that were also commonly used phrases were removed from the businesses list. Radiology clinics, medical laboratories and physiotherapy clinics were removed from the businesses list and placed in the healthcare facilities list. Municipalities and businesses were split into 'ambiguous' and 'unambiguous' by cross referencing against all of the other lists. Those that also appeared on other lists were placed into 'ambiguous' lists while all others remained on the 'unambiguous' lists.

To the number string searches we added modifications to detect Canadian postal codes (letter number letter number letter number) and Ontario healthcard numbers (a string of 10 numbers that may or may not be separated into groups of 4, 3 and 3 digits plus or minus one, or two letter version codes). To remove medical record numbers we removed numbers that were between 5 and 9 digits. In addition, the ethnicity, international cities and local places lists that were part of the original *deid *program were also removed either for irrelevance or in order to preserve clinical context.

#### Names

The existing *deid *program name removal is based on both a dictionary look up for known patient and provider names and context checks. This dictionary look up is akin to our 'initial name removal' phase that is performed at the physician office before data is transferred to ICES. For the context checks it groups names into names that are either, 'ambiguous', 'unambiguous' or 'popular.' 'Ambiguous' names are only removed if they occur beside a first name or a last name, or if there is an immediate word preceding or following such as 'Dr.', 'Mr.', 'daughter', 'mother', 'husband', etc. For the 'ambiguous' names list we used the existing list and added a separated list of first names and last names from the Registered Persons Database (RPDB) which records the first and last names of all residents in the province of Ontario and includes over 700,000 unique last names. We also incorporated a list of nicknames[23] developed by a team of researchers at the University of Ottawa to the 'ambiguous' name list. 'Unambiguous' names are removed with every occurrence in the text. To this list we added additional names that were not included in the original list and were used as the replacement names for the preliminary name de-identification that occurred in the physician office prior to data transfer. The original *deid *'popular' names list was not altered and names that were in the 'popular' names list and also on the other name lists were removed from the other lists. Names that were on both the 'unambiguous' and 'ambiguous' names list were removed from the 'ambiguous' names list. For physician names we used a separated list of first and last names from the 2006 Canadian Medical Directory[24].

After the lists were created, all except the 'unambiguous' names list were checked against a list of common English words that came with the original *deid *program and any names or words that were also commonly used English words were removed so as to prevent removal of words that were a part of phrases describing clinical information. The 'unambiguous' names was checked against a shorter list of the most common English words that also came with the original *deid *program, in order to detect the names that were also one of the most common English names and if the name was on the list it was similarly removed.

#### Protecting medical eponyms

Medical eponyms are diseases, syndromes, signs or symptoms that are named after someone, often the physician or person that first described or discovered it, or a patient that was afflicted with it. Some examples include Parkinson's disease, Homan's sign and Apgar score. All are common terms used in the medical realm but to an automated de-identification system, may appear to be a person and then erroneously removed. Although *deid *had a short list of around 25 medical eponyms to check names and words that were identified as potentially identifying we expanded this by including 600 more chosen from a list of over 6000 medical eponyms[25]. These 600 were the most commonly used, as determined by the family physician/investigator (KT).

#### Further modifications and the creation of a 'do not remove' list

After running the program initially, several recurrent program errors were identified. Thus we added coding to prevent the removal of single letters followed by punctuation. This was done to protect single letter short forms such as the acronym S O A P standing for subjective, objective, assessment and plan, a commonly used format for physicians to record clinical encounters. We also added coding in order to prevent removal of typical medical nomenclature, that could be mistaken for a postal code, such as maternal pregnancy history depicted as G2P1A1 denoting gravida of 2, parity of 1 and abortions 1, C6C7T1 denoting cervical spine 6, cervical spine 7 and thoracic spine 1, or S1S2S4 nomenclature denoting heart sounds. The program also removed words such as Operative-Smith considering it to be a hyphenated last name, thus we modified the program to prevent this error. Last, we modified the context street address part of the program requiring a number followed by a word for all Drive's to be removed as the program was erroneously removing phrases such as, 'fitness to drive.'

After these further modifications were made, there were still recurrent errors identified necessitating the creation of a 'do not remove' list. This list of words and phrases was used to over-ride decisions made by *deid*. It included the countries and states from the original *deid *program to preserve ethnicity and travel that may have clinical implications. As well, it included medications, common medical acronyms (ie.1 mm ST elevation as typically used in descriptions of electrocardiograms), parts of the body and words that are commonly used in a clinical context. The list of medications was taken from the Ontario Drug Benefit formulary. The list of body parts and systems was derived from the Canadian Classification of Health Interventions (CCI), and the list of common words and phrases was created after reviewing the list of words that were deemed false positive or negative and recognizing common words or phrases that were being removed such as assessment and emergency.

### Performance measures used

We report sensitivity/recall as the percentage of positive labeled instances of PHI that were predicted as positive, specificity as the percentage of negative (unlabeled) instances that were predicted as negative, precision (or positive predictive value) as the percentage of positive predictions that were correct and accuracy as the percentage of predictions that were correct. F- measure, a combination of precision and recall with equal weighting, was measured using the formula: F-measure = 2(precision × recall)/(precision + recall).

This project received ethics approval from Sunnybrook Health Sciences Centre Research Ethics Board.

## Results

The original *deid *program, prior to any modifications, performed with a similar precision but more than 10% lower recall on our data compared to the performance on US nursing notes reported by the originators showing 74.9% precision and 96.7% recall[17]. Modifying the program and replacing US locations and business lists with Ontario ones resulted in an improvement in recall while the addition of our name lists did not make further substantial improvements. Adding the medical eponyms list had no impact and adding the protection for common acronyms and nomenclature resulted in minimal improvements. However, adding the 'do not remove' lists greatly improved the specificity, precision, accuracy and F-measure while only slightly decreasing the recall. (see Table 1)

**Table 1:** Results of the original *deid *program and modified program on the training set and two validation sets

Feature added/Modified	Number of Free Text Records	Sensitivity/Recall	Specificity	Precision	Accuracy	F-measure
**Original *deid *Program**	**500**	**83.4%**	**71.6%**	**71.0%**	**77.0%**	**0.77**

**Modification of *deid *Program**						

- Replaced *deid *lists for cities, businesses and medical facilities with Ontario lists and made adjustments for Ontario healthcard numbers and postal codes	**500**	**91.5%**	**71.0%**	**70.7%**	**79.9%**	**0.80**

- Added RPDB* names to ambiguous names, added PS‡ derived initial name removal replacement names to the unambiguous names and added list of Ontario physicians	**500**	**90.9%**	**71.8%**	**71.5%**	**80.1%**	**0.80**

- Improved medical eponyms lists	**500**	**90.9%**	**71.8%**	**71.5%**	**80.1%**	**0.80**

- Added protection for common acronyms and nomenclature	**750**	**92.6%**	**72.8%**	**72.7%**	**81.5%**	**0.81**

- Added 'do not remove' list	**1000**	**88.3%**	**91.4%**	**91.3%**	**89.9%**	**0.90**

**First Validation**	**700**	**86.7%**	**91.4%**	**91.1%**	**89.0%**	**0.89**

**Second Validation**	**500**	**80.2%**	**87.7%**	**87.4%**	**83.8%**	**0.84**

*RPDB = Registered Persons Database

‡PS = Practice Solutions

When the final modified program was run on the validation sets, the sensitivity and accuracy dropped but the specificity and precision was similar to that in the final training set thereby supporting the generalizability of the modified program while protecting clinical content. (see Table 1)

## Discussion

We found that *deid *could be modified to fit and work on free-text primary care EMR data in Ontario, Canada. However major modifications to the program were necessary to bring the specificity, precision and accuracy up to an acceptable level in order to prevent loss of clinical information in the de-identification process.

While other de-identification programs report very high recall (> 95%) on defined types of medical free-text documents such as discharge summaries[11,13-15] or pathology reports,[8-10,16] our results are inclusive of all types of free-text records contained in primary care EMRs including point-form progress notes, diagnostic tests, operatives reports, consultation letters and discharge summaries. Furthermore, our data was real world data from multiple different geographic locations, with text from multiple types of physicians and allied health professionals. The aim of other programs have been for maximal recall whereas not overzealously replacing words or phrases that may have clinical relevance was of greater importance to us. Additionally, our data goes through a first pass name removal and then instances of PHI are replaced by pseudonyms that are generated by our modified program. To a reader, missed occurrences of PHI that are not replaced are difficult to detect as they are mixed in with the pseudonym PHI.

Adaptation of *deid *to Swedish[26] and French[27] has been generally unsuccessful. Both attempts were confined to hospital records and both had challenges of adaptation into a different language with different grammatical rules. It was not surprising to find that both groups found *deid *to cause over-deidentification similar to what we found in the English language until we created a 'do not remove' list consisting of common over de-identification terms that was a result of the context portion of the *deid *program.

Admittedly there are number of limitations to our modified *deid *program that affect the generalizability of this tool. First, the main de-identification process occurs at ICES to allow for in-house analysis of data. ICES given its 'prescribed entity' status and designated data covenanters that are permitted to handle identifiable personal level health information and therefore can partition the data and run the de-identification software, is a relatively unique situation not only in Canada but also the rest of the world. Thus application of this software in other jurisdictions may not be sufficient to meet general privacy standards. However, as the development of 'one patient one record' programs develop in other provinces and other countries the findings here are likely to contribute to this growing field. Second, this program was developed on Practice Solutions^® ^EMR software with its unique data structure and layout leading to a tendency for physicians to insert data into their EMR in an optical character recognition (OCR) format which renders the text searchable and editable. Not all EMR data from other vendors is structured this way and often in other EMRs external paper documents are scanned in, captured and stored in a picture like format such as a tiff, pdf, or jpeg. Documents that are stored in this manner need to be further processed and converted to an OCR format before de-identification can occur and doing so can distort words or introduce typographical errors that can be missed by an automated system looking for matching words. Third, to train and test our program, we took a sample of records from all of our current data and although our validation set results were comparable to our training set results, it is possible that not all ways of entering data were captured in our random sample. New physicians that we add to the database may have their own unique style of entering data and this may lead to errors in our de-identification processing. Last, given the large additional lists we have added to the program, de-identification of documents is time consuming taking approximately 47 hours to process 5 years of data on 2900 patients.

## Conclusion

Despite these limitations we now have a reasonably accurate tool for de-identifying primary care EMR data in EMRALD. Future research could focus on developing efficient tools to de-identify data at the extraction point and prior to transfer. Nonetheless this tool will be applied to all of our free-text EMR data currently existing in our database and future data that will be collected. This greatly facilitates the use of all of the valuable information contained in the free-text fields of the EMR which will allow for a more maximal use of EMR data not impeded by patient privacy and confidentiality issues.

## Competing interests

The authors declare that they have no competing interests.

## Authors' contributions

All of the authors were involved in the conception and design of the study. JM led the initial conceptual approach to the de-identification of free-text EMR data. MC was primarily responsible for the program modifications, with additional conceptual contributions from JK, KT and TM. KT and JK prepared the first draft of the article, and KT was responsible for subsequent revisions. All of the authors critically reviewed the article and approved the final version.

## Funding

Data acquisition was supported by a grant from the Canadian Institutes of Health Research (CIHR) to the Canadian Cardiovascular Outcomes Research Team (CCORT) grant FRN:CTP79847. The National Research Council of Canada provided the support for the initial conceptual design and students. Finally, the analysis was supported by a grant from the Ontario Ministry of Health and Long-Term Care Enhancing Quality in Primary Care.

This study was supported by the Institute for Clinical Evaluative Sciences (ICES), which is funded by an annual grant from the Ontario Ministry of Health and Long-Term Care (MOHLTC). The opinions, results and conclusions reported in this paper are those of the authors and are independent from the funding sources. No endorsement by ICES or the Ontario MOHLTC is intended or should be inferred.

## Pre-publication history

The pre-publication history for this paper can be accessed here:

http://www.biomedcentral.com/1472-6947/10/35/prepub
